# A machine learning framework supporting prospective clinical decisions applied to risk prediction in oncology

**DOI:** 10.1038/s41746-022-00660-3

**Published:** 2022-08-16

**Authors:** Lorinda Coombs, Abigail Orlando, Xiaoliang Wang, Pooja Shaw, Alexander S. Rich, Shreyas Lakhtakia, Karen Titchener, Blythe Adamson, Rebecca A. Miksad, Kathi Mooney

**Affiliations:** 1 Huntsman Cancer Institute, University of Utah, Salt Lake City, UT USA; 2 Flatiron Health, Inc, New York, NY USA; 3 Present Address: University of North Carolina-Chapel Hill, Lineberger Cancer Institute, Chapel Hill, NC USA

**Keywords:** Health care, Risk factors

## Abstract

We present a general framework for developing a machine learning (ML) tool that supports clinician assessment of patient risk using electronic health record-derived real-world data and apply the framework to a quality improvement use case in an oncology setting to identify patients at risk for a near-term (60 day) emergency department (ED) visit who could potentially be eligible for a home-based acute care program. Framework steps include defining clinical quality improvement goals, model development and validation, bias assessment, retrospective and prospective validation, and deployment in clinical workflow. In the retrospective analysis for the use case, 8% of patient encounters were associated with a high risk (pre-defined as predicted probability ≥20%) for a near-term ED visit by the patient. Positive predictive value (PPV) and negative predictive value (NPV) for future ED events was 26% and 91%, respectively. Odds ratio (OR) of ED visit (high- vs. low-risk) was 3.5 (95% CI: 3.4–3.5). The model appeared to be calibrated across racial, gender, and ethnic groups. In the prospective analysis, 10% of patients were classified as high risk, 76% of whom were confirmed by clinicians as eligible for home-based acute care. PPV and NPV for future ED events was 22% and 95%, respectively. OR of ED visit (high- vs. low-risk) was 5.4 (95% CI: 2.6–11.0). The proposed framework for an ML-based tool that supports clinician assessment of patient risk is a stepwise development approach; we successfully applied the framework to an ED visit risk prediction use case.

## Introduction

The advent of widespread electronic health record (EHR) implementation coincided with healthcare advances that rapidly increased the volume and complexity of information clinicians can access about individual patients. Yet, from a clinical data synthesis perspective, the EHR and other electronic sources of clinical information still hold a wealth of untapped information that can benefit overall patient care. Unlocking the promise of electronically-stored healthcare data to improve healthcare across a population of patients requires better development and application of tools that collect and synthesize digitally stored data^1^.

The use of real-world data (RWD) from sources such as EHRs, registries, and claims data for the development of machine learning (ML)-based predictive risk models is an emerging research area^2–9^. However, to date the vast majority of research about the utility and value of ML approaches in the healthcare setting has been retrospective in nature, used historical data for model development and validation, and was limited to specific use cases^10^. There is a notable lack of prospective evaluation of ML tools in healthcare, which has hindered their widespread adoption into real-world clinical workflows in an evidenced-based fashion^10,11^. Indeed, in a recent systematic literature review of studies that used ML tools to address a clinical problem, just 2% of reviewed studies were prospective in design^10^. Additionally, there is a need for a consistent ML model evaluation framework in order to standardize approaches and facilitate comparisons across tools, data sources, and use cases.

The use of ML-based tools to aid the preemptive identification of patients who are at risk for an adverse clinical event could improve overall patient care and safety through more efficient healthcare delivery and the prompting of an early intervention to mitigate severity. The overall objective of this paper is to put forth a general framework to evaluate and deploy an ML-based clinical tool that supports a clinician’s independent assessment of patient risk for an adverse event by displaying medical information and predicted risk level using documented EHR-derived RWD. Then, in order to demonstrate the functionality and utility of the framework, we present an example use case in the oncology setting.

## Results—use case

### Retrospective evaluation

The retrospective evaluation included 28,433 encounters (2385 patients); 53% were women, the median age was 65 years, and 87% were White (Table 1). The most common cancers (excluding non-melanoma skin neoplasms) were breast, unspecified primary malignant neoplasms, prostate, and non-Hodgkin's lymphoma as defined by standard ICD mapping rules. The observed prevalence of one or more ED visit(s) within 60 days was 10% and the ML-based clinical tool classified 8% of encounters as high risk (pre-defined predicted probability ≥0.20). The positive predictive value (PPV) and negative predictive value (NPV) for future ED events was 26% and 91%, respectively. Patients identified as high risk by the tool had 3.5 times greater odds of a 60-day ED visit than those identified as low risk (95% CI: 3.4–3.5; Table 2).

**Table 1:** Baseline patient characteristics for model training and retrospective evaluation.

Characteristics	Categories	Model training cohort *N* = 5139 patients	Retrospective cohort *N* = 2385 patients
Age, years	Median (range)	64 (18–100)	65 (18–101)
Gender	Male	47%	47%
	Female	53%	53%
Ethnicity	Hispanic	7%	7%
	Non-Hispanic	93%	93%
Race	White	88%	87%
	Black	1%	1%
	Asian	2%	2%
	Other	8%	8%
	Unknown	1%	2%
Cancer Sites^a^	Breast	24%	23%
	Unspecified primary malignant neoplasms	22%	21%
	Non-melanoma skin neoplasms	20%	22%
	Prostate	15%	16%
	Lung	8%	10%
	Multiple myeloma	7%	8%
	Non-Hodgkin’s lymphoma	11%	11%
	Leukemia	8%	9%
	Colon	6%	6%
	Melanoma of skin	9%	8%
	Bone/connective tissue	8%	9%
	Brain	<5%	5%
	Head and neck	6%	6%

^a^Only cancer sites with ≥5% prevalence at baseline are listed in the table.

**Table 2:** Retrospective and prospective evaluation results.

Model performance metric	Retrospective result	Prospective result
ED prevalence	10%	7%
Predicted risk level, proportion of patients classified as “high risk”	8%	10%
Sensitivity (sens) [aka: recall]	19% (95% CI: 19–20)	32% (95% CI: 18–48)
Specificity (spec)	93% (95% CI: 93–93)	92% (95% CI: 90–94)
PPV	26% (95% CI: 26–26)	22% (95% CI: 12–34)
NPV	91% (95% CI: 91–91)	95% (95% CI: 93–97)
OR of ED visit (high-risk vs. low-risk patients)	3.5 (95% CI: 3.4–3.5)	5.4 (95% CI: 2.6–11.0)

Prospective evaluation metrics are at the patient level and retrospective evaluation metrics are calculated at the encounter level.

*ED* emergency department, *NPV* negative predictive value, *PPV* positive predictive value.

### Bias assessment

**Fig. 1:**
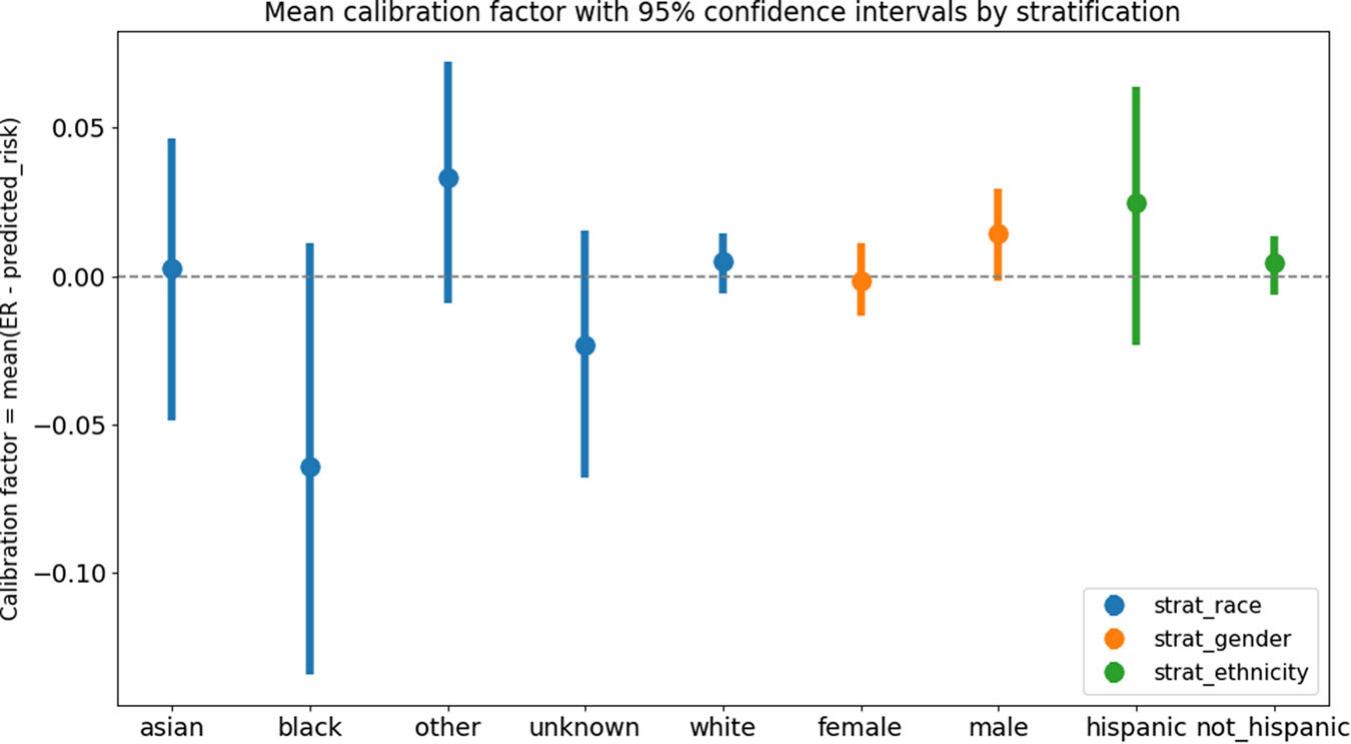
Calibration factor, by stratification. Calibration factor with 95% confidence intervals, stratified on different race, gender and ethnicity values.

The observed calibration factors for the majority groups in the datasets were as follows: White race: 0.005 [−0.006, 0.014]; Female gender: −0.001 [−0.013, 0.011]; Non-Hispanic ethnicity: 0.004 [−0.006, 0.013] (Table 3). Patient demographic groups constituting minorities along the lines of race, gender, and ethnicity are also reported in Table 3. In all cases, 0 laid within the bootstrapped 95% confidence interval for the metric (i.e., ideal calibration). In the analysis, using calibration factors to assess the model’s fairness performance, the model appeared to be largely calibrated across racial, gender, and ethnic groups, although, as noted previously, the relevant point estimates for some groups have wide confidence intervals (Fig. 1).

**Table 3:** Calibration factor results.

Group type	Group	Estimated calibration factor [95% confidence interval]
Ethnicity	Hispanic	0.024 [−0.023, 0.064]
	Not Hispanic	0.004 [−0.006, 0.014]
Gender	Female	−0.001 [−0.013, 0.011]
	Male	0.014 [−0.002, 0.029]
Race	Asian	0.003 [−0.048, 0.046]
	Black	−0.064 [−0.134, 0.011]
	Other	0.033 [−0.009, 0.072]
	Unknown	−0.023 [−0.068, 0.015]
	White	0.005 [−0.006, 0.015]

### Prospective evaluation

The prospective evaluation included 1236 patients; 53% were women, the median age was 65 years, and 84% were White (Table 4). The most common cancers (excluding non-melanoma skin cancer) were breast, prostate, lung, and multiple myeloma. The observed prevalence of an ED visit within 60 days was 7%. The ML-based clinical tool classified 10% of patients as high risk; of these higher risk patients, 76% were confirmed by a Huntsman at Home nurse practitioner review of the EHR that their clinical course would qualify them for admission to the Huntsman at Home program (95% CI: 0.62–0.89). The PPV and NPV for future ED events was 22% and 95%, respectively. Patients identified as high risk by the tool had 5.4 times greater odds of a 60-day ED visit than those identified as low risk (95% CI: 2.6–11.0; Table 2).

**Table 4:** Baseline characteristics among Huntsman patients with cancer in the prospective validation study.

Characteristics	Categories	Hold out cohort *N* = 633			Deliverable cohort *N* = 603		
		Overall	High risk *n* = 61	Low risk *n* = 572	Overall	High risk *n* = 44	Low risk *n* = 559
Age (years)	Median	65	63	65	65	64	65
	Mean (range)	63 (20–95)	62 (29–95)	63 (20–95)	63 (20–93)	62 (28–88)	63 (20–93)
Gender, *n* (%)	Male	300 (47)	27 (44)	273 (48)	278 (46)	24 (55)	254 (45)
	Female	333 (53)	34 (56)	299 (52)	325 (54)	20 (45)	305 (55)
Ethnicity, *n* (%)	Hispanic	51 (8)	8 (13)	43 (8)	41 (7)	5 (11)	36 (6)
	Non-Hispanic	582 (92)	53 (87)	529 (92)	562 (93)	39 (89)	523 (94)
Race, *n* (%)	White	529 (84)	49 (80)	480 (84)	512 (85)	33 (75)	479 (86)
	Black	10 (2)	1 (2)	9 (2)	10 (2)	3 (7)	7 (1)
	Asian	15 (2)	3 (5)	12 (2)	16 (3)	2 (5)	14 (3)
	Other	66 (10)	8 (13)	58 (10)	52 (9)	6 (14)	46 (8)
	Unknown	13 (2)	0 (0)	13 (2)	13 (2)	0 (0)	13 (2)
Medicaid, *n* (%)	Yes	76 (12)	28 (46)	48 (8)	63 (10)	16 (36)	47 (8)
	No	557 (88)	33 (54)	524 (92)	540 (90)	28 (64)	512 (92)
H@H enrollment at index encounter, *n* (%)	Yes	20 (3)	9 (15)	11 (2)	26 (4)	9 (20)	17 (3)
	No	613 (97)	52 (85)	561 (98)	577 (96)	35 (80)	542 (97)
Number of cancer diagnosis, *n* (%)	1	309 (49)	28 (46)	281 (49)	295 (49)	17 (39)	278 (50)
	2	175 (28)	13 (21)	162 (28)	150 (25)	14 (32)	136 (24)
	3	80 (13)	7 (11)	73 (13)	75 (12)	6 (14)	69 (12)
	4	31 (5)	6 (10)	25 (4)	40 (7)	4 (9)	36 (6)
	≥5	38 (6)	7 (11)	31 (5)	43 (7)	3 (7)	40 (7)
Cancer Sites^a^, *n* (%)	Breast	129 (20)	3 (5)	126 (22)	129 (21)	2 (5)	127 (23)
	Unspecified primary malignant neoplasms	123 (19)	18 (30)	105 (18)	152 (25)	14 (32)	138 (25)
	Non-melanoma skin neoplasms	111 (18)	9 (15)	102 (18)	117 (19)	6 (14)	111 (20)
	Prostate	95 (15)	8 (13)	87 (15)	83 (14)	6 (14)	77 (14)
	Lung	63 (10)	11 (18)	52 (9)	66 (11)	5 (11)	61 (11)
	Multiple myeloma	56 (9)	2 (3)	54 (9)	70 (12)	8 (18)	62 (11)
	Non-Hodgkin’s lymphoma	54 (9)	6 (10)	48 (8)	65 (11)	5 (11)	60 (11)
	Leukemia	53 (9)	7 (11)	46 (8)	57 (9)	3 (7)	54 (10)
	Colon	46 (7)	11 (18)	35 (6)	31 (5)	5 (11)	26 (5)
	Melanoma of skin	42 (7)	5 (8)	37 (6)	45 (7)	3 (7)	42 (8)
	Bone/connective tissue	40 (6)	5 (8)	35 (6)	43 (7)	4 (9)	39 (7)
	Kidney/renal pelvis	35 (6)	6 (10)	29 (5)	26 (4)	1 (2)	25 (4)
	Rectum	33 (5)	9 (15)	24 (4)	21 (3)	2 (5)	19 (3)
	Ovary	31 (5)	3 (5)	28 (5)	28 (5)	4 (9)	24 (4)
	Uterus	30 (5)	2 (3)	28 (5)	22 (4)	3 (7)	19 (3)
	Brain	29 (5)	4 (7)	25 (4)	26 (4)	3 (7)	23 (4)
	Head and neck	27 (4)	4 (7)	23 (4)	34 (6)	4 (9)	30 (5)

^a^Only cancer sites with >5% prevalence at baseline are listed in the table.

*H@H* Huntsman at Home Program.

We also conducted exploratory stratified analysis by encounter dates before or after 01-13-2020 (60 days prior to national response date on 03-13-2020^12^) to assess whether the model performance was affected by the impact of COVID-19 on patient behaviors and healthcare delivery. The outcome forecast precision (PPV) was lower after the date cut-off chosen to identify patient encounters whose 60 day “at risk” period overlapped the starting of the pandemic.

## Discussion

We have developed a standardized framework to evaluate and deploy an ML-based clinical tool that supports a clinician’s independent assessment of patient risk for adverse clinical events by displaying medical information and predicted risk level using documented EHR-derived RWD. As a collaborative multidisciplinary team, we tested the ML-based clinical tool retrospectively on a representative target patient population, evaluated it for potential algorithmic bias, and—importantly—piloted its use in a prospective healthcare system quality improvement study that demonstrated clinically-useful model performance.

Several important learnings were derived from the development of the framework. A key takeaway was that prospective validation (framework step 5) was important for gaining clinician trust in the output of the ML-based adverse event risk prediction tool, which is critical for successful implementation of ML tools in a healthcare setting^13,14^. In our use case, results of the prospective validation did not indicate additional model changes were necessary for clinical usefulness beyond that suggested in the retrospective analysis. This finding raises the question of when prospective validation of an ML model is useful for deploying the model in different settings, for different patient populations, or for different endpoints. When previous prospective evaluation of the same (or a very similar) model was reassuring in a generally similar population, we believe appropriate rigorous prospective, ongoing monitoring of performance and adequate education to ensure user trust in the model are appropriate guardrails. However, it should be noted that this is speculative based on our experience deploying the ML model prospectively, and additional evaluations of ML-based tools in clinical practice can help to clarify the settings for which prospective research evaluation is necessary.

Additionally, groups responsible for assessing ML models to improve health outcomes should weigh both the upfront resources required for model development and validation, as well as the ongoing resources needed to responsibly monitor changes in performance and bias over time once embedded in the clinical workflow^14^. In the early stage of model development, it is critical to have upfront engagement from the clinical team who will utilize the tool, as well as to seek clinical feedback throughout the whole process. Discussions with all stakeholders on the practicality and clinical impact of each step will help to prioritize resources and steer the focus of model development. As we demonstrated in the use case, the ML-based clinical tool was focused on increasing the efficiency of surfacing high-risk patients to clinical staff in a timely manner, compared to no information provided by the ML-based clinical tool. Therefore, although the model did not perfectly predict all patients who truly had a 60-day ED visit, the PPV was largely improved compared to the baseline prevalence of risk. Additionally, the choice of modeling approach should be determined early on in the model development process. In this study, random forest and logistic regression models were both tested in the prototype phase. While the random forest model demonstrated slightly better performance than the logistic regression model (Supplementary Table 1), the logistic regression model was chosen for the prospective evaluation because it was better suited to the use case needs (i.e., easily interpretable and communicated to all users, including clinical partners involved in its development). Any modeling choice is accompanied by tradeoffs. In using a logistic regression, our model had high transparency and parsimony, but slightly lower performance. This could be because a logistic regression does not automatically capture interactions amongst features as a tree-based model can. Such tradeoffs between model performance, interpretability, and feature interactions and exhaustiveness should be considered in the context of the use case and different models should be tested prior to carrying out prospective evaluations using the final model.

There are few published articles that present a standardized framework or guidance for developing ML-based tools in a healthcare setting^11,13,14^, and we were unable to find any that included a prospective evaluation of the framework in a real-world use case study. Our proposed framework has several areas of strength. It is comprehensive, standardized, and versatile in its adaptability to various diseases and healthcare data types (e.g., EHR, claims, or registry data). A key strength is the pre-specification of the ML model design and evaluation plan. Additionally, the framework differentiates between retrospective and prospective validation steps, and it highlights the utility of a pilot program prior to full implementation, as demonstrated through our use case. Importantly, the framework emphasizes the necessity of bringing together a multidisciplinary team to develop the ML-tool collaboratively and iteratively. Integrating technical knowledge with clinical knowledge helps to break down information silos between team members. This approach improves human explainability of the ML model, so that all involved have a strong understanding of the tool in order to best serve patients, which is critical for early acceptance and broad adoption.

There are limitations to consider regarding the use of any ML tool in a healthcare setting^14^. First, ML models are not perfect tools for predicting the risk of an adverse clinical event. Accordingly, they should be used to support clinician assessment, not to replace it, and transparency is critical. Additionally, care would need to be taken to preserve model fairness and interpretability. In our use case, the PPV of 22% in the prospective study leaves an opportunity for improvement of the model's predictive performance. Potential directions for improvement of model performance include introducing additional clinical features to the model such as those extracted from unstructured clinical text, and exploring more sophisticated, nonlinear ML techniques. Second, an ML-based clinical tool may not include all possible clinical factors that may be predictive of the defined clinical event. Sensitivity analysis should be conducted during the retrospective evaluation to determine whether the inclusion and exclusion of certain factors affect the model performance. Third, an inherent limitation of using EHR-derived RWD to develop an ML-based clinical tool is the potential for missingness in the dataset; data not documented in the dataset cannot be taken into consideration by the risk model. Fourth, the model performance and real-world utility may vary with the time period of assessment. Our exploratory analysis stratified by COVID response date suggests that different factors (or a different impact of existing model factors) may improve model performance for near-term ED visits in the COVID setting. These performance changes can sometimes be mitigated through rigorous monitoring and model recalibration or retraining^15,16^; this updating could be more difficult in the face of large, sudden changes such as a pandemic. Fifth, there might be a trade-off between computational efficiency, practical usability, and clinical relevance. The ML-based tool demonstrated in this use case was built using retrospective encounter-level data and applied to weekly encounter-level data for computational efficiency and workflow fluency during clinical practice. However, the tool was prospectively evaluated using patient-level data for model validation and clinical impact on patients. Therefore, it is important to evaluate the ML-based tool from various aspects throughout the whole process. Finally, while the framework outlined in this paper is generalizable, the resulting algorithm may not be. Specific analyses and data used to build the model and analyze the performance should be localized to the specific healthcare setting in which it will be implemented. For instance, this model was trained using only data from patients from the HCI, and is thus applicable prospectively to that setting. Should this framework be used to create solutions for other patient populations, the steps of the framework should be repeated using target data from the relevant population and any models should be customized to meet the unique and specific objectives of the healthcare system.

In conclusion, we have developed a general framework to evaluate and deploy an ML-based clinical tool that supports a clinician’s independent assessment of patient risk and applied it in a real-world oncology setting retrospectively and prospectively. Future additional applications of the framework should be explored and can help to inform and refine approaches to developing ML tools for prospective use in healthcare settings.

## Methods

In the following sections, the steps of the proposed ML-based clinical tool evaluation framework are described, followed by the methods for application of the framework to a healthcare system quality improvement use case at a large cancer center.

### ML-based clinical tool evaluation general framework steps

The ML-tool evaluation general framework was developed by a multidisciplinary team of software engineers, data scientists, clinicians, researchers, administrators, and managers. Each step of the framework along with considerations is outlined in Table 5. In order to obtain value from the framework and to prevent wasted efforts, the following assumptions should be considered: (1) pursuit of the defined clinical quality improvement goal will meaningfully enhance patient care while maintaining or improving the efficiency and sustainability of healthcare delivery; (2) prediction of the defined clinical event enables care teams to make progress towards this goal; and (3) the ML-based clinical tool can be implemented in a way that improves technology utilization rather than contributing to provider burn-out.

**Table 5:** ML-based clinical tool evaluation framework steps.

**Step 1: Define clinical quality improvement goal and opportunity unlocked by predicting the clinical event**	
*Considerations*	*Impact*
• Define patient care/quality goals • Identify actionable clinical events that if predicted help achieve goals • Establish metrics and results required to identify “at risk” patients • Evaluate if this type of tool is useful for furthering goals • Determine how the tool will embed into clinical workflow, and what actions need to be taken based on predicted clinical event • Define key metrics for evaluating clinical impact of risk predictions	All stakeholders (clinicians, business leaders, data scientists, etc.,) will have a clear understanding of how deployment of an ML-based clinical tool will help to achieve quality improvement goals. Teams should be able to fill in this statement: “If the care team knows that X event will happen, they will take Y action, to increase Z value”.
**Step 2: Build/acquire ML-based clinical tool that predicts defined clinical event**	
*Considerations*	*Impact*
Decide whether to build a custom ML-based tool or acquire an existing ML-based tool that is practical, customizable, and suited for the practice’s local data patterns	Organization will be equipped with the right ML-based clinical tool for their intended goals
**Step 3: Conduct retrospective evaluation of ML-based clinical tool**	
*Considerations*	*Impact*
Retrospectively apply model to a representative historical patient population from the institution and then compare predictions with known past observed events to confirm if the tool meets desired metrics	Allows the organization to expediently assess the suitability of the ML-based clinical tool for the prediction task at hand
**Step 4: Conduct bias assessment**	
*Considerations*	*Impact*
• Proactively evaluate for bias, including treatment pattern disparities or lack of representation, choice of modeling approach, or choice of predicted clinical event • Make necessary adjustments to the tool before there is any impact on patients	Ensures that the ML-based clinical tool algorithms do not reproduce real-world inequalities that can occur as a result of treatment pattern disparities or a lack of representation encoded in datasets, the choice of modeling approach, or the choice of predicted clinical event
**Step 5: Conduct prospective evaluation of ML-based tool**	
*Considerations*	*Impact*
• Conduct a prospective evaluation on a present-day, real-world patient population in a randomized setting to understand how well the model is likely to perform in real time • Note: This step may not be necessary in every case if the ML-based tool has been prospectively evaluated and its performance in real-world setting monitored	Prospective validation is considered the “gold standard” of ML model validation when applied to the point-of-care setting because it shows the clinical team how well the model is likely to perform in real time where several factors can affect model performance, such as recent pattern changes in the real world (e.g., occurrence of a pandemic), care delivery (e.g., updates to clinical standards), or technical or operational issues (e.g., data entry delays that can make a system unusable in practice)
**Step 6: Embed and monitor tool in clinical workflow**	
*Considerations*	*Impact*
• Adopt tool into standard clinical workflow • Conduct data quality monitoring, performance monitoring, and bias monitoring • The ML-based tool should not replace traditional patient identification processes, but support them with a data-driven approach that also enhances their efficiency	The ML-based tool can now be used to achieve the quality improvement goal defined in Step 1. Ongoing monitoring ensures the model’s suitability in the dynamic clinical environment of the real world where patterns of care seeking and care delivery evolve, and that model predictions are not impacted by manual or technical errors that could inadvertently affect a patient’s predicted risk and/or access to supplemental care.

*ML* machine learning.

### Application of the framework to an oncology use case

To demonstrate the steps described in the general framework, we applied it to a real-world oncology setting. Together, the Huntsman Cancer Institute and Flatiron Health evaluated whether a Flatiron Health-developed supplemental ML-based clinical tool and monitoring framework could support the clinical program staff at the Huntsman Cancer Institute to enhance identification of potentially eligible patients for Huntsman at Home, a program providing acute level “hospital at home” care along with palliative and hospice services^17^. We followed best practices for model transparency and validation and the minimum information about clinical artificial intelligence modeling (MI-CLAIM) checklist is provided in Supplementary Table 2^18,19^. This study complies with all relevant ethical regulations. The study protocol was submitted to the University of Utah Institutional Review Board (IRB_00127233), which determined that this quality improvement project did not meet the definitions of Human Subjects Research according to Federal regulations and therefore IRB oversight was not required or necessary for the study.

### Use case step 1: Define healthcare system quality improvement goal met by predicting clinical event

A key healthcare system quality improvement goal for the Huntsman Cancer Institute is to reduce emergency department (ED) and hospital utilization for patients with cancer. To make progress towards this goal, the Huntsman Cancer Institute established the home-based Huntsman at Home program in 2018, which provides acute-level care to patients with cancer for conditions that commonly require ED evaluation and/or rehospitalization, such as poorly controlled symptoms, pain, dehydration, or infections. Episodic palliative or supportive care visits and/or end of life hospice care are also provided. The intent of this suite of comprehensive services is to improve patient quality of life, lengthen time at home, reduce avoidable ED visits and hospitalization stays, and improve family caregiver well-being^17,20^. Patients considered for enrollment to the Huntsman at Home program are identified by two pathways, direct clinician referral or as part of a hospital discharge plan either at the end of a stay or as part of an early discharge pathway. Since a large volume of unplanned hospitalizations occur through the ED, Huntsman at Home was interested in proactively identifying patients who were likely to have a near-term ED visit (60-day ED visit).

After aligning on key goals outlined in Step 1 of the framework, we were able to state the following: If the Huntsman at Home team anticipates that a patient who is being treated for cancer at Huntsman Cancer Institute may have a near-term ED visit, they will enroll the patient in Huntsman at Home, which will improve the patient experience and reduce the total cost of care.

### Use case step 2: Build or acquire ML-based clinical tool that predicts defined clinical event

The ML-based clinical tool development was led by a multidisciplinary team at Flatiron Health. The probability of near-term ED visit was estimated based on demographic and clinical characteristics, using a logistic regression model with L2 regularization. We also tested model performance in the retrospective analysis (described in Step 3 below) using a random forest model in order to compare performance of the two models (Supplementary Table 1). The outcome of interest was narrowly defined in service of the broader quality improvement objective in Step 1. In defining the outcome of interest, care was taken to avoid any measurement error that could come from using proxies, as discussed in work by Mullainathan and Obermeyer^21^. Thus, near-term ED visits captured directly through the EHR were chosen as the model’s outcome of interest. In addition, this step presents as an opportunity to assess what qualitative factors such as model explainability, transparency, and fairness should be considered.

Demographic and clinical features were pre-determined based on Flatiron Health oncology data expertise and informed by Huntsman at Home clinical experience, and the final feature list included in the model was determined based on the data quality and feature importance (Supplementary Table 3). Demographic features included gender, race/ethnicity, and history of Medicaid enrollment. Clinical features included cancer diagnosis, comorbidities, lab test results for albumin, bilirubin, hematocrit and hemoglobin, weight loss, recency and frequency of prior visits, and prior medication orders and clinic administrations.

The model was trained on all patient encounters with the University of Utah Health System (e.g., office visits, diagnostic visits, emergency visits) from 01-01-2016 to 12-31-2018 using cross-validation to select model hyperparameters (Supplementary Material Notes). Prediction estimates were calculated only for patients who met the inclusion/exclusion criteria (Supplementary Table 4), (based on Huntsman at Home program guidelines) at the time of the index encounter for which a prediction was made. The model produces risk scores for visits, which represent the predicted probability of a subsequent ED visit by that patient within 60 days of the visit. A separate validation set of patients was used to assess initial model performance and to set a risk threshold such that visits with risk scores above it would be classified as “high risk”. A pragmatic risk threshold was chosen tailored to the Huntsman at Home program size and patient review capacity. A predicted probability of 0.20 was selected so that 10% of all visits were classified as high risk in order to make the manual effort associated with weekly review of predictions feasible for clinical experts in the Huntsman at Home program. The same risk threshold of 0.20 was applied in the prospective validation study (described below).

The model was trained on individual encounters, and thus included multiple observations per patient. This methodological decision was driven by the use case in which repeated risk assessments were needed over time because the patients' risk factors may change relatively quickly. Since the goal of the ML-based clinical tool was to predict an updated risk score for each eligible patient following any clinical interaction with the University of Utah Health System, we developed the model to capture a diversity of patient states along their care journeys. In other words, training a model on all encounters (e.g., office visits, diagnostic visits, emergency visits) meant that the team was able to learn from information that was gained between encounters (e.g., an updated lab result). This choice also necessitated the use of temporal features (e.g., patient had a visit to the ED in the 30 days before the current encounter). Including temporal features allowed the model to account for the recency of certain information, which was important because we made new predictions for each patient encounter over time rather than one prediction for all time. In determining what features were best suited to being coded temporally and what time bins to create for them, we consulted with clinical experts at Flatiron Health and HCI and performed a literature review. For example, we created binary features for albumin lab test results being abnormal in each of the recent windows (e.g., 5, 30, 90 days leading up to the encounter) in order to account for both very recent changes (e.g., what happened in the last 5 days before an encounter) that could elevate patient risk, as well as more long term features (e.g., 90 days) that could set a temporal baseline.

### Use case step 3: Conduct retrospective evaluation of ML-based clinical tool

After determining that use of a logistic regression model with L2 regularization best suited the use case needs, we retrospectively evaluated the ML-based clinical tool on an independent test set of 28,433 samples representing encounters at the University of Utah Health System from 03-01-2019 to 09-30-2019 for the 2,385 patients who met the inclusion/exclusion criteria (Supplementary Table 4). Testing on data not used to train the model is common in ML to prevent model overfitting. Baseline metrics were calculated to describe the test set and included age (median [minimum–maximum; IQR]), gender (female, male), ethnicity (Hispanic/Latino), race (White, Asian, Black, Other), documented Medicaid payer prior to the index encounter date, and cancer diagnosis (categorical with categories based on ICD codes mapped to cancer groupings as per standard Flatiron Health data processing procedures^22,23^). An exploratory post-hoc analysis assessed model accuracy with standard metrics, as defined in Table 6.

**Table 6:** Definitions of metrics to assess model accuracy.

Metrics	Definition
ED prevalence (%)	Prevalence of observed 60-day ED visit: proportion (0–100%)
Predicted risk level	Binary, high/low; proportion (high risk %)
Sensitivity (sens) [aka: recall]	Proportion of encounters classified as high risk among those with ED visit
Specificity (spec)	Proportion of encounters classified as low risk among those without ED visit
Positive predictive value	Proportion of encounters followed by an ED visit among those classified as high risk
Negative predictive value	Proportion of encounters without a subsequent ED visit among those classified as low risk
Odds ratio	Odds ratio of ED visit among high-risk encounters vs. low-risk encounters

*ED* emergency department.

### Use case step 4: Conduct bias assessment of ML-based clinical tool

Different definitions of fairness within the context of racial/ethnic bias and ML have been proposed, including anti-classification, classification parity of specific metrics, and calibration (Corbett-Davies and Goel)^24^. Calibration measures the gap between predicted risk for an outcome and observed outcomes for a given group across different levels of predicted risk. An ideally fair model from this perspective would be one where this gap is non-existent for all groups of interest. Based on precedent in the literature for problems of risk prediction, such as the analysis in Obermeyer et al.^25^ we mitigated bias through assessment of calibration fairness. To assess calibration fairness, we produced calibration curves that compared, by group, the predicted risk of utilizing the ED against the observed rate of doing so. Using the same test set that was used to evaluate model performance, we also calculated a summary statistic for each group, a “calibration factor” that measured the difference between the mean predicted risk and the mean observed outcome for a group^26^. Models satisfying calibration fairness should have a calibration factor of zero (Supplementary Material Notes). We reviewed overall calibration curves for indications of subtler forms of bias that might not be captured by the calibration factor. In addition to avoiding measurement error and assessing the model for calibration fairness, we minimized other risks of bias by choosing transparent and interpretable modeling techniques (e.g., logistic regression). This was done to preclude other unforeseeable harm that could be a by-product of black box techniques^27^.

### Use case step 5: Conduct prospective evaluation of ML-based clinical tool

**Fig. 2:**
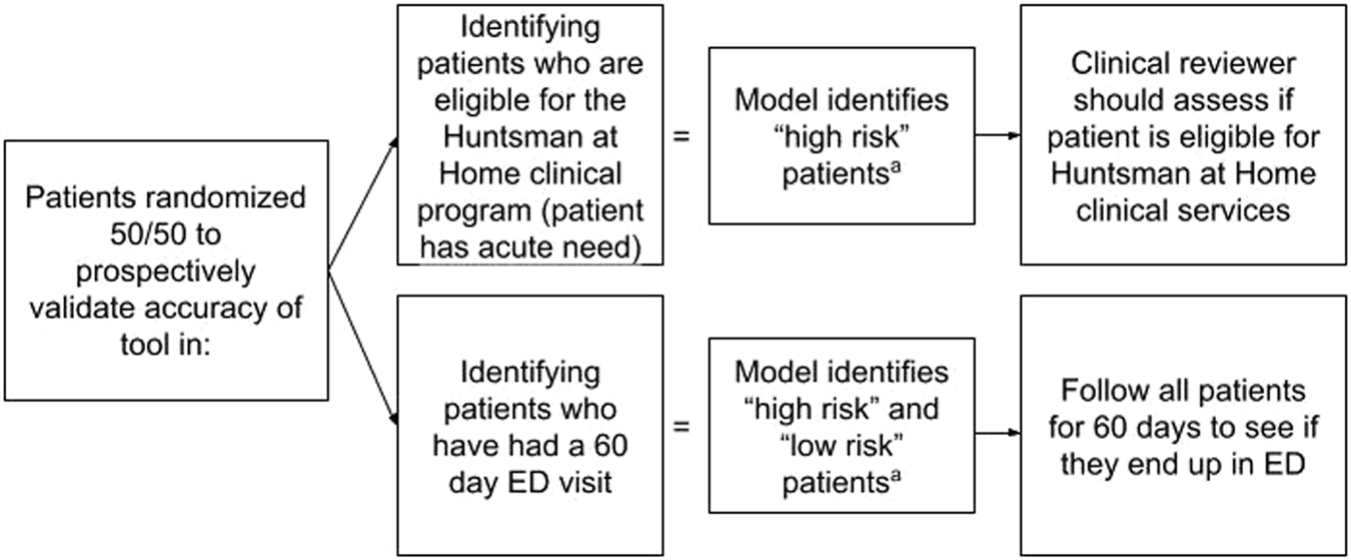
Prospective evaluation randomization approach. ED emergency department. ^a^If the patient’s predicted probability of risk is greater than the classification threshold the patient will be classified as “high risk”, otherwise the patient will be classified as “low risk”. The classification threshold is selected using retrospective data prior to the start of the prospective evaluation.

Given the additional goal of evaluating impact on patient outcomes prospectively in the clinical setting, we assessed model performance at the patient level. A prospective quality improvement study of 1236 patients was conducted to evaluate the accuracy and the usability of this ML-based model among patients with a visit to the Huntsman Cancer Institute between 01-04-2020 and 02-07-2020. Otherwise applying the same inclusion/exclusion criteria as the retrospective portion of the study, patients were randomly selected, in a 1:1 ratio, to be in either the independent “hold out” sub-cohort to assess model accuracy, or the “deliverable” sub-cohort to assess real-world accuracy (Fig. 2). Patient-level index encounter was defined as the encounter that corresponded to the first high-risk classification for high-risk patients and first encounter for low-risk patients to ensure that each observation was statistically independent and capable of yielding valid, clinically meaningful inferences. Each patient was followed up for 60 days after the index encounter. We assessed model accuracy in the “hold-out” cohort using the metrics summarized in Table 6.

To evaluate prediction accuracy of the risk stratification model, we compared patients with predicted risk classification against their observed outcomes of ED visit within 60 days of the index encounter. For real-world accuracy, the clinical expert reviewed the charts of patients who were identified as high-risk by the risk stratification model and were in the “deliverable” cohort to classify their eligibility for the Huntsman at Home program based on Huntsman at Home standard protocol. We estimated the risk model’s clinical accuracy by the proportion of eligible patients among all the identified high-risk patients in the “deliverable” cohort. Baseline characteristics were described for both the “hold-out” and “deliverable” sub-cohorts to evaluate the success of randomization.

### Use case step 6: Embed and monitor ML-based clinical tool in clinical workflow

Based on successful outcomes from preceding steps, the Huntsman at Home team is operationalizing the ML-based clinical tool, which will be used to supplement traditional referral pathways (e.g., clinician referrals) to the Huntsman at Home program by displaying relevant information. Once a patient is surfaced for evaluation (from any source), all decisions for Huntsman at Home enrollment are made by the clinical care team, based on standardized clinical evaluation processes, and not solely on the risk prediction result from the tool. Transparency was ensured through clear documentation and the clinician could independently review the basis of the risk prediction. To support the ML-based clinical tool in production, Flatiron Health developed a solution that continuously monitors the ML-based clinical tool predictions to ensure that the algorithms developed on retrospective data do not lead to unanticipated outcomes in a prospective real-world setting.

### Reporting summary

Further information on research design is available in the Nature Research Reporting Summary linked to this article.

## Supplementary information

Reporting Summary

Supplementary Material

## Data Availability

The de-identified data that support the findings of this study may be made available upon request, and are subject to a license agreement; interested researchers should contact <DataAccess@flatiron.com> to determine licensing terms.

## Code availability

The custom computer code used to generate the results of this study is available to the journal editors and study peer reviewers upon request for purposes of validating the results of the study. Because aspects of the custom computer code used to generate results reported in the paper is the proprietary confidential information of Flatiron Health, it cannot be made publicly available.
