# Supplementary material for: A machine learning framework supporting prospective clinical decisions applied to risk prediction in oncology
Source: NPJ Digit Med. 2022 Aug 16;5:117. doi: 10.1038/s41746-022-00660-3 (PMC9380664; doi:10.1038/s41746-022-00660-3)
Supplement: Supplementary file 1 — Reporting Summary [file 41746_2022_660_MOESM1_ESM.pdf]

Corresponding author(s): Rebecca Miksad
DBPR NPJDIGITALMED-02933R1

Last updated by author(s): Jul 7, 2022

Reporting Summary

Nature Portfolio wishes to improve the reproducibility of the work that we publish. This form provides structure for consistency and transparency in reporting. For further information on Nature Portfolio policies, see our Editorial Policies and the Editorial Policy Checklist.

Statistics

For all statistical analyses, confirm that the following items are present in the figure legend, table legend, main text, or Methods section.

n/a	Confirmed	
☐	☒	The exact sample size (*n*) for each experimental group/condition, given as a discrete number and unit of measurement
☐	☒	A statement on whether measurements were taken from distinct samples or whether the same sample was measured repeatedly
☒	☐	The statistical test(s) used AND whether they are one- or two-sided<br>*Only common tests should be described solely by name; describe more complex techniques in the Methods section.*
☒	☐	A description of all covariates tested
☒	☐	A description of any assumptions or corrections, such as tests of normality and adjustment for multiple comparisons
☒	☐	A full description of the statistical parameters including central tendency (e.g. means) or other basic estimates (e.g. regression coefficient) AND variation (e.g. standard deviation) or associated estimates of uncertainty (e.g. confidence intervals)
☒	☐	For null hypothesis testing, the test statistic (e.g. *F*, *t*, *r*) with confidence intervals, effect sizes, degrees of freedom and *P* value noted<br>*Give P values as exact values whenever suitable.*
☒	☐	For Bayesian analysis, information on the choice of priors and Markov chain Monte Carlo settings
☒	☐	For hierarchical and complex designs, identification of the appropriate level for tests and full reporting of outcomes
☒	☐	Estimates of effect sizes (e.g. Cohen's *d*, Pearson's *r*), indicating how they were calculated

Our web collection on statistics for biologists contains articles on many of the points above.

Software and code

Policy information about availability of computer code

Data collection	Clinical characteristics of patients in the University of Utah Health system were collected in an electronic health record system in the course of clinical care and were processed using proprietary Flatiron Health code.
Data analysis	The data was analyzed using proprietary Flatiron Health code using the python and R programming languages.

For manuscripts utilizing custom algorithms or software that are central to the research but not yet described in published literature, software must be made available to editors and reviewers. We strongly encourage code deposition in a community repository (e.g. GitHub). See the Nature Portfolio guidelines for submitting code & software for further information.

Data

Policy information about availability of data

All manuscripts must include a data availability statement. This statement should provide the following information, where applicable:

- Accession codes, unique identifiers, or web links for publicly available datasets
- A description of any restrictions on data availability
- For clinical datasets or third party data, please ensure that the statement adheres to our policy

The de-identified data that support the findings of this study may be made available upon request, and are subject to a license agreement; interested researchers should contact <DataAccess@flatiron.com> to determine licensing terms.

Field-specific reporting

Please select the one below that is the best fit for your research. If you are not sure, read the appropriate sections before making your selection.

☒ Life sciences ☐ Behavioural & social sciences ☐ Ecological, evolutionary & environmental sciences

For a reference copy of the document with all sections, see nature.com/documents/nr-reporting-summary-flat.pdf

Life sciences study design

All studies must disclose on these points even when the disclosure is negative.

Sample size	The sample size was not predetermined. Rather, the retrospective evaluation of the ML-based clinical tool included all patient encounters at the University of Utah Health system from 03-01-2019 to 09-30-2019 for the 2,385 patients who met the inclusion/exclusion criteria. The prospective evaluation included all 1236 patients with a visit to the Huntsman Cancer Institute between 01-04-2020 and 02-07-2020.
Data exclusions	Patient inclusion/exclusion criteria for retrospective and prospective evaluation of the ML-based clinical tool are described within the manuscript. No additional data exclusions were applied.
Replication	The study includes a retrospective evaluation of the ML-based clinical tool, and a subsequent prospective evaluation that (1) confirms the ability of the tool to identify patients at high risk of an ED visit, and (2) confirms that patients identified as high risk by the tool are judged eligible for the Huntsman at Home program.
Randomization	Patients in the prospective evaluation were allocated randomly to one of two groups. The "hold-out" group was used to evaluate algorithm accuracy at predicting ED visit, and the "delivery" group was used to evaluate eligibility of predicted patients for Huntsman at Home clinical services.
Blinding	Blinding was not relevant to this study, which involved application and evaluation of a machine learning algorithm.

Reporting for specific materials, systems and methods

We require information from authors about some types of materials, experimental systems and methods used in many studies. Here, indicate whether each material, system or method listed is relevant to your study. If you are not sure if a list item applies to your research, read the appropriate section before selecting a response.

Materials & experimental systems

n/a	Involved in the study	
☒	☐	Antibodies
☒	☐	Eukaryotic cell lines
☒	☐	Palaeontology and archaeology
☒	☐	Animals and other organisms
☒	☐	Human research participants
☐	☒	Clinical data
☒	☐	Dual use research of concern

Methods

n/a	Involved in the study	
☒	☐	ChIP-seq
☒	☐	Flow cytometry
☒	☐	MRI-based neuroimaging

Clinical data

Policy information about clinical studies

All manuscripts should comply with the ICMJE guidelines for publication of clinical research and a completed CONSORT checklist must be included with all submissions.

Clinical trial registration	This study was not a clinical trial, it was an observational study using de-identified real world data.
Study protocol	Institutional Review Board approval of the study protocol was obtained prior to study conduct, and included a waiver of informed consent.<br>Protocol number and title: RWE-001: The Flatiron Health Real World Evidence Parent Protocol<br>IRB name and registration number: WCG IRB; IRB00000533.<br>Protocol approval ID, tracking number: 420180044.
Data collection	We retrospectively evaluated the ML-based clinical tool on an independent test set of 28,433 samples representing encounters at the University of Utah Health System from 03-01-2019 to 09-30-2019 for the 2,385 patients who met the inclusion/exclusion criteria.<br><br>A prospective quality improvement study of 1236 patients was conducted to evaluate the accuracy and the usability of this ML-based model among patients with a visit to the Huntsman Cancer Institute between 01-04-2020 and 02-07-2020.

Outcomes	A near-term emergency department visits captured directly through the EHR were chosen as the model's outcome of interest. Near term was defined as an ED visit within 60 days of the index encounter.
