# Supplementary material for: A machine learning framework supporting prospective clinical decisions applied to risk prediction in oncology
Source: NPJ Digit Med. 2022 Aug 16;5:117. doi: 10.1038/s41746-022-00660-3 (PMC9380664; doi:10.1038/s41746-022-00660-3)
Supplement: Supplementary file 2 — Supplementary Material [file 41746_2022_660_MOESM2_ESM.pdf]

## Supplementary Material

### *Supplemental Notes:*

*Details of machine learning feature generation and algorithm*

Each sample used for the machine learning (ML) model training represented a single patient visit to a University of Utah facility. The label predicted for that sample was defined as 1 if the patient visited a University of Utah emergency department (ED) within 60 days of the visit, and 0 otherwise.

Features (covariates) used by the model were selected through a collaborative and iterative process involving clinical experts and data scientists. An initial list of potential features was informed by input from clinicians, review of existing literature, and data scientists who evaluated which of these features were accessible in structured electronic health record (EHR) data and had acceptable completeness and prevalence in conjunction with clinical experts.

All features used in the model were structured as binary variables. A list of features can be found in Supplemental Table 3. We included both time-varying and non-time-varying features as inputs to the model in order to accommodate the longitudinal nature of patient health and potential deterioration. This enables the model to learn relationships between not just clinical concepts, and to account for their recency in relation to ED visits. Some features, such as race, ethnicity, and sex, were assumed not to change across all visits for a given patient. Others, such as diagnoses, were considered present for all visits on or after the appearance of the relevant ICD code. Features related to past visits, medications, or changes in vitals made use of clinically-relevant time

windows relative to the visit of interest. Examples of time window features include whether the patient received an antiemetic order in the past 30 days, and whether the patient had lost greater than 5 pounds in the past 30 days.

The ML model was a L2-regularized logistic regression. We used k-fold cross-validation with 5 folds, using the AUC metric to select the best-performing level of regularization.

*Calibration factor definition*

We used calibration factor as a summary statistic to evaluate the calibration within each group of interest. The calibration factor was defined as follows. For each recorded visit *i* within a group of interest, the model calculates a risk score $r_i$ between 0 and 1. We then observe an outcome $o_i$, either ED utilization or no ED utilization, encoded as 1 or 0 respectively. The calibration factor is then $cf = mean(r_i) - mean(o_i)$ $cf=mean(r_i)-mean(o_i)$ calculated over all visits and outcomes in that group. A calibration factor greater than 0 indicates that the model is over-predicting risk within the group, while a calibration factor less than 0 indicates that the model is under-predicting risk within the group.

*Supplemental Tables*

*MI-CLAIM Checklist.*

After the framework in this study was developed and the use cases were designed and developed, an external group of leaders in the healthcare machine learning community published a guide for transparent reporting, The Minimum Information about Clinical Artificial Intelligence Modeling (MI-CLAIM) Checklist. We have included the checklist completed here (Supplemental Table 1) with our post-hoc assessment of the manuscript as a supplement to promote transparency.

***Supplemental Table 1: Comparison of Models as Part of Retrospective Analysis***

| **Model performance metric** | **Logistic Regression (actual model used) Retrospective result** | **Random Forest (model hyperparameters tuned using random grid search) for comparison** |
|---|---|---|
| ED prevalence observed | 10% | 10% |
| Model AUC | 0.69 | 0.72 |
| Model threshold | 0.20 | 0.20 |
| Predicted risk level, proportion of patients classified as “high risk” | 8% | 9% |
| Sensitivity (sens) [aka: recall] | 19% (95% CI: 19–20) | 23% (95% CI: 23–23) |
| Specificity (spec) | 93% (95% CI: 93–93) | 93% (95% CI: 93–93) |
| PPV | 26% (95% CI: 26–26) | 26% (95% CI: 26–26) |
| NPV | 91% (95% CI: 91–91) | 91% (95% CI: 91–91) |
| OR of ED visit (high-risk vs low-risk patients) | 3.5 (95% CI: 3.4–3.5) | 3.7 (95% CI: 3.7–3.8) |

Note: Prospective evaluation metrics are at the patient level and retrospective evaluation metrics for both models are calculated at the encounter level.

Abbreviations: ED, emergency department; AUC, area under the (receiver operating characteristic) curve;

NPV, negative predictive value; PPV, positive predictive value.

*Supplemental Table 2. The MI-CLAIM checklist*[a]

| **Study design (Part 1)** | **Completed: page number** | **Notes if not completed** |
|---|---|---|
| The clinical problem in which the model will be employed is clearly detailed in the paper. | Yes; pg. 13 | |
| The research question is clearly stated. | Yes; pg. 12,13 | |
| The characteristics of the cohorts (training and test sets) are detailed in the text. | Yes; pg. 14-16 | |
| The cohorts (training and test sets) are shown to be representative of real-world clinical settings. | Yes; pg. 14-16 | |
| The state-of-the-art solution used as a baseline for comparison has been identified and detailed. | Other leading modeling approaches were considered at the time of model building, some results are in Supplemental Table 1 | |
| **Data and optimization (Parts 2, 3)** | **Completed: page number** | **Notes if not completed** |
| The origin of the data is described and the original format is detailed in the paper. | Yes; pg. 15-16, 18-19 | |
| Transformations of the data before it is applied to the proposed model are described. | Yes; pg. 16 | Due to space constraints and IP protection, a full provenance of the data is omitted from the manuscript, most relevant methods are described on pg. 16 |
| The independence between training and test sets has been proven in the paper. | Yes; pg. 17 | |
| Details on the models that were evaluated and the code developed to select the best model are provided. | Yes; pg. 14-17 | Note: The de-identified data that support the findings of this study may be made available upon request, and are subject |

| | | |
|---|---|---|
| | | to a license agreement; interested researchers should contact <DataAccess@flatiron.com> to determine licensing terms. |
| Is the input data type structured or unstructured? | Structured | |
| **Model performance (Part 4)** | **Completed: page number** | **Notes if not completed** |
| The primary metric selected to evaluate algorithm performance (e.g., AUC, F-score, etc.), including the justification for selection, has been clearly stated. | Yes; pg. 14-19 | |
| The primary metric selected to evaluate the clinical utility of the model (e.g., PPV, NNT, etc.), including the justification for selection, has been clearly stated. | Yes; pg. 17-19 | |
| The performance comparison between baseline and proposed model is presented with the appropriate statistical significance. | Yes, Supplemental Table 1 | |
| **Model examination (Part 5)** | **Completed: page number** | **Notes if not completed** |
| Examination technique 1a | Yes; model examination was completed using coefficients and sensitivity analysis; these can be made available upon request. | |
| Examination technique 2a | See above | |
| A discussion of the relevance of the examination results with respect to model/algorithm performance is presented. | Yes; pg. 7-11 | |
| A discussion of the feasibility and significance of model interpretability at the case level if examination methods are uninterpretable as presented. | Model methods used were interpretable; see discussion | |

| | | |
|---|---|---|
| | on pg. 8-9, 16 | |
| A discussion of the reliability and robustness of the model as the underlying data distribution shifts is included. | Yes; pg. 10-11 | |
| **Reproducibility (Part 6): choose appropriate tier of transparency** | **N/A** | **Notes** |
| Tier 1: complete sharing of the code | | |
| Tier 2: allow a third party to evaluate the code for accuracy/fairness; share the results of this evaluation | | |
| Tier 3: release of a virtual machine (binary) for running the code on new data without sharing its details | | |
| Tier 4: no sharing | ✓ | Note: The de-identified data that support the findings of this study may be made available upon request, and are subject to a license agreement; interested researchers should contact <DataAccess@flatiron.com> to determine licensing terms. |

MI-CLAIM, Minimum information about clinical artificial intelligence modeling; N/A, not applicable.
[a]Source: Norgeot B, Quer G, Beaulieu-Jones BK, et al. Minimum information about clinical artificial intelligence modeling: the MI-CLAIM checklist. *Nat Med*. 2020;26(9):1320-1324. doi:10.1038/s41591-020-1041-y

***Supplemental Table 3. Example Model Features***

| Category | Example Features[a] |
|---|---|
| Demographics | age brackets ≥55 years, ≥65 years, ≥75 years, ≥85 years, gender, medicaid insurance effective ever, hispanic ethnicity, race (asian, black, unknown, other) |
| Labs | hemoglobin, hematocrit, bilirubin in range or out of range for 5 day, 30 day and 90 day time periods |
| Comorbidities | cardiac disease, HIV/AIDS, hepatitis, Autism |
| Primary Cancer Diagnosis | Brain cancer, Lung cancer, Breast cancer |
| Secondary Malignant Neoplasms | Secondary malignant neoplasm of left adrenal gland, Secondary and unspecified malignant neoplasm of lymph node unspecified |
| Visits or Admissions | 1, 2 or 3+ visits/admission to ED, inpatient, ICU within past 5, 30, or 90 days |
| Medications | antiemetic, anti-depressant, pain medication, anti-neoplastic order in last 5, 30, or 90 days |
| Vitals | change of 5%, 10%, 20% of body weight or more |

Abbreviations: ED, emergency department; ICU, intensive care unit.
[a]This is not the full list of features used in the model.

***Supplemental Table 4. Inclusion/Exclusion Criteria***[a]

| | |
|---|---|
| Inclusion Criteria | a. Age 18 years or older at the date of the University of Utah encounter<br>b. Has a cancer diagnosis ICD code dated prior to the encounter at the University of Utah<br>c. Has at least one University of Utah encounter during the study time period<br>i. Model training time period: 01-01-2016 to 12-31-2018<br>ii. Retrospective study time period: 03-01-2019 to 09-30-2019<br>iii. Prospective study time period: 01-04-2020 to 02-07-2020 |
| Exclusion Criteria | a. Insufficient structured oncology data: first ever oncology-related visit at the University of Utah is less than 90 days prior to the prediction encounter at the University of Utah (i.e., patients newly diagnosed with a cancer ICD code within 90 days prior to the prediction encounter will be excluded)<br>b. Inactive patient: has fewer than two oncology-related visits in the 90 days prior to the prediction encounter(s) at the University of Utah<br>c. Ineligible zip code for enrollment at Huntsman at Home: zip code is not within the 20-mile radius of the University of Utah (eligible zip code list provided by Huntsman at Home team) |

ICD, International Classification of Diseases
[a]The inclusion/exclusion criteria were the same for model training and the retrospective and prospective studies (with the exception of eligibility time period).
